# Transcriptional regulation of autophagy in skeletal muscle stem cells

**DOI:** 10.1242/dmm.052007

**Published:** 2025-02-10

**Authors:** Priya D. Gopal Krishnan, Wen Xing Lee, Kah Yong Goh, Sze Mun Choy, Lewin Raymarc Roldan Turqueza, Zhuo Han Lim, Hong-Wen Tang

**Affiliations:** ^1^ Program in Cancer and Stem Cell Biology, Duke-NUS Medical School, 8 College Road, Singapore 169857, Singapore; ^2^ Division of Cellular and Molecular Research, Humphrey Oei Institute of Cancer Research, National Cancer Centre Singapore, Singapore 169610, Singapore

**Keywords:** Autophagy, Muscle regeneration, Muscle stem cells, Muscle diseases

## Abstract

Muscle stem cells (MuSCs) are essential for the regenerative capabilities of skeletal muscles. MuSCs are maintained in a quiescent state, but, when activated, can undergo proliferation and differentiation into myocytes, which fuse and mature to generate muscle fibers. The maintenance of MuSC quiescence and MuSC activation are processes that are tightly regulated by autophagy, a conserved degradation system that removes unessential or dysfunctional cellular components via lysosomes. Both the upregulation and downregulation of autophagy have been linked to impaired muscle regeneration, causing myopathies such as cancer cachexia, sarcopenia and Duchenne muscular dystrophy. In this Review, we highlight the importance of autophagy in regulating MuSC activity during muscle regeneration. Additionally, we summarize recent studies that link the transcriptional dysregulation of autophagy to muscle atrophy, emphasizing the dominant roles that transcription factors play in myogenic programs. Deciphering and understanding the roles of these transcription factors in the regulation of autophagy during myogenesis could advance the development of regenerative medicine.

## Teaser

**Summary:** This Review highlights recent studies linking transcriptional dysregulation of autophagy to muscle stem cell dysfunction. A deeper understanding of autophagy regulation in muscle stem cells could facilitate the development of regenerative therapies for muscle atrophy-related diseases.

## Introduction

Skeletal muscle comprises 40-50% of the total body mass and is a vital organ essential for mechanical locomotive and metabolic activities (Han et al., 2022; Sartori et al., 2021). Skeletal muscle is composed of multinucleated cells called myofibers, which form from the fusion of myoblasts. Myoblasts differentiate from muscle stem cells (MuSCs), also known as satellite cells (Relaix et al., 2021). MuSCs are located beneath the basal lamina of myofibers and, unlike post-mitotic muscle cells, can undergo proliferation and differentiation. This ability allows them to maintain their pool of progenitors and to give rise to differentiated myocytes, which fuse with each other or with existing myofibers to regenerate muscles (Chen and Goldhamer, 2003).

MuSCs primarily exist in a quiescent state, and are defined by the expression of specific antigens, such as CD34 and M-cadherin (also known as CDH15), and of transcription factors, such as the paired homeobox factors, PAX3 and PAX7, which are crucial for MuSC maintenance and self-renewal (Pang et al., 2023). These transcription factors control the expression of genes that repress differentiation while maintaining the proliferative capacities of MuSCs (Almada and Wagers, 2016). Upon injury, MuSCs become activated and exit quiescence, a process that is mediated by myogenic regulatory factors, including MyoD (also known as MYOD1), myogenic regulatory factor 4 (MRF4) and myogenic factor 5 (MYF5) (Hung et al., 2023). This transient amplifying population of myogenic progenitors, known as myoblasts, migrates to sites of injury. Upon arrival, they receive localized signals that trigger their differentiation into myocytes, which are characterized by increased expression of myogenin and myosin heavy chain (MHC). These mature myocytes can then fuse at an injury site to repair damaged muscles (Relaix et al., 2021).

**Fig. 1.**
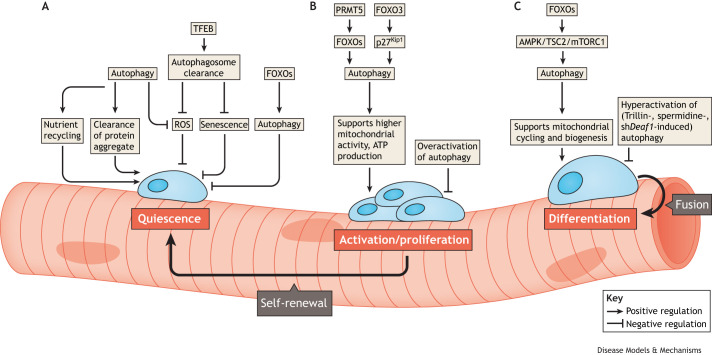
**Summary of the roles of autophagy in maintaining homeostasis in muscle stem cells (MuSCs).** (A) In quiescence, autophagy supports MuSC homeostasis by recycling nutrients and clearing protein aggregates. TFEB activates lysosomal and autophagy genes to prevent senescence and ROS buildup, protecting MuSCs. By contrast, FOXOs regulate autophagy components such as LC3 and BNIP3, leading to premature MuSC activation and impaired regeneration. (B) During MuSC activation and proliferation, the PRMT5–FOXO pathway enhances mitochondrial activity and ATP production through autophagy. FOXO3-driven cytoplasmic expression of p27^Kip1^ promotes autophagy and MuSC proliferation, but overactive autophagy can inhibit proliferation. (C) During MuSC differentiation into myotubes, autophagy is essential to meet energy demands. The FOXO–AMPK–TSC2–mTORC1 pathway drives autophagosome formation, which is critical for muscle regeneration. However, excessive autophagy from treatments such as Trillin, spermidine or sh*Deaf1* can impair differentiation. AMPK, AMP-activated protein kinase; ATP, adenosine triphosphate; FOXO, Forkhead box class O; mTORC1, mammalian target of rapamycin complex 1; PRMT5, protein arginine methyltransferase 5; ROS, reactive oxygen species; sh*Deaf1*, short hairpin RNA against deformed epidermal autoregulator factor-1; TFEB, transcriptional factor EB; TSC2, tuberin.

A critical regulator of skeletal muscle regeneration is autophagy, an evolutionarily conserved lysosome-dependent catabolic pathway that degrades unwanted cellular components and that provides cells with protein and energy (Han et al., 2022; Tang et al., 2013). Autophagy plays fundamental roles in maintaining cellular balance in MuSCs (Fig. 1). For instance, autophagy is essential for the removal of damaged mitochondria, thus preventing the production of harmful reactive oxygen species (ROS) that are detrimental to MuSCs (Mortensen et al., 2011; Wang et al., 2022) (Fig. 1). Autophagy also prevents senescence and genome instability by protecting cells against potential metabolic stress (García-Prat et al., 2016) (Fig. 1). Additionally, autophagy is crucial for all processes of myogenesis (see Glossary, Box 1), including quiescence, activation and differentiation of MuSCs, through epigenetic and metabolic regulation (Ho et al., 2017), highlighting the important roles that autophagy fulfils in muscle regeneration.

**Box 1. Glossary**

**3-Methyladenine (3-MA):** an autophagy inhibitor that blocks autophagy by preventing autophagosome formation through the inhibition of type III phosphatidylinositol 3-kinases.

**Autophagy flux:** a measure of autophagic degradation activity.

**Bafilomycin A1 (Baf-A1):** an autophagy inhibitor that prevents autophagosomes and lysosomes from fusing by selectively inhibiting vacuolar H^+^ ATPase.

**Cachexia:** a condition that leads to significant loss of muscle mass and weight in patients with cancer and other chronic diseases.

**Chloroquine:** an antimalarial drug that inhibits autophagy flux by impairing autophagosome–lysosome fusion.

**Duchenne muscular dystrophy (DMD):** a genetic disorder characterized by progressive muscle loss.

**Extracellular vehicles:** membrane-bound organelles secreted by cells into the extracellular space.

**Hyperglycemia:** a condition in which the blood glucose level is higher than normal.

**mTORC1–TFEB signaling pathway:** a signaling pathway by which mTORC1 and the TFEB transcription factor interact through TFEB phosphorylation to coordinate metabolic supply.

**Muscle atrophy:** a condition characterized by a reduction in muscle mass.

**Myogenesis:** a multi-step process that refers to the formation and development of muscular tissue from undifferentiated cells.

**Myomixer:** a micropeptide that is muscle specific and aids the transmembrane protein myomaker to modulate embryonic myoblast fusion and muscle formation.

**Myotonic dystrophy type 1 (DM1):** a multi-system disorder that primarily leads to progressive muscle loss, weakness and myotonia.

**Proteostasis:** a process that regulates proteins within cells to maintain the health of the cellular proteome and the organism itself.

**Pompe disease:** also known as glycogen storage disease type II, a metabolic disorder that creates glycogen deposits inside lysosomes within the muscle tissue.

**Sarcopenia:** age-related loss of muscle mass and strength.

In recent years, the pivotal role of autophagy in skeletal muscle stem cell biology has gained significant attention owing to its implications for muscle regeneration and aging. This Review focuses specifically on the role of autophagy in MuSCs, emphasizing its importance in maintaining cellular homeostasis and promoting muscle repair. By focusing on MuSCs, we aim to provide a detailed understanding of how transcription factors regulate autophagic processes and how these processes influence muscle stem cell quiescence, activation and differentiation. For broader perspectives on autophagy in other muscle cell types and metabolic pathways, we refer readers to recent comprehensive reviews (e.g. Chen et al., 2022; Han et al., 2022), as these topics fall outside the primary scope of this article.

## Physiological functions of autophagy in MuSCs

Autophagy flux (Box 1) changes during muscle regeneration. By removing excess or damaged organelles and proteins, or by regulating intracellular oxidative stress signals, autophagy contributes to myogenic differentiation and enhances muscle recovery (Baechler et al., 2019; Call and Nichenko, 2020). However, both impaired and excessive autophagy can lead to muscle atrophy (Box 1), highlighting the need for strict regulation of autophagy during muscle regeneration, as we discuss in this section.

### Quiescence

Under normal conditions, MuSCs remain in quiescence, a state of low cellular activity in which the limited energy demands of MuSCs are met by a low metabolic rate. This quiescent state allows MuSCs to be preserved as a reserve for future regenerative needs (Ancel et al., 2021). Owing to their limited energy demands, proteostasis (Box 1) is strictly controlled in MuSCs, and the suppression of protein translation, via the inhibition of the translation initiation factor eIF2α, is observed in quiescent MuSCs (Zismanov et al., 2016). When eIF2α is mutated, this repression fails and translation is initiated, activating the myogenic program and leading to the precocious differentiation and subsequent loss of MuSCs (Zismanov et al., 2016).

In addition to translation repression, basal levels of autophagy are essential for the maintenance of MuSC quiescence. Autophagy facilitates the recycling and clearance of protein aggregates, preventing cellular damage. In *Atg7^ΔPax7^* mice, which carry MuSC-specific deletion of autophagy related 7 (*Atg7*), the gene that encodes an essential protein for autophagosome formation, autophagy is abolished, leading to senescence and to the loss of quiescent MuSCs in muscle tissue (García-Prat et al., 2016). Autophagy loss also results in mitochondrial dysfunction and in the accumulation of organelles, proteins and ROS. Pharmacological inhibition of ROS, through the treatment of aged LC3–GFP mice with Trolox (a vitamin E analogue), prevents senescence and restores the self-renewal capacity of these aged autophagy-deficient MuSCs (García-Prat et al., 2016). These findings underscore the necessity of autophagy for the maintenance of MuSC quiescence and regenerative potential.

The AMP-activated protein kinase (AMPK; also known as PRKA) signaling pathway is a key regulator of autophagy in MuSCs. In aged MuSCs purified from old mice, the decreased phosphorylation of AMPK and its target p27^Kip1^ (also known as CDKN1B) results in suppressed autophagy and in increased apoptosis, rendering aged MuSCs highly susceptible to cell death when autophagy is impaired (White et al., 2018). Activation of the AMPK/p27^Kip1^ pathway by re-treating old MuSCs with the AMPK activator, 5-aminoimidazole-4-carboxamide ribonucleotide (AICAR), enhanced autophagy and reduced senescence markers, offering potential therapeutic strategies with which to maintain MuSC quiescence and prevent apoptosis in aged or damaged muscles (White et al., 2018). Together, these studies emphasize the importance of autophagy and AMPK signaling in preserving MuSC function and muscle regeneration.

### Activation and proliferation of MuSCs

In response to stimuli, such as muscle injury or exercise, MuSCs exit quiescence to become activated. They then re-enter the cell cycle to proliferate and generate muscle progenitor cells. Some activated and proliferative muscle progenitor cells differentiate and fuse to form new myofibers for muscle repair, while a subpopulation of progenitor cells undergoes self-renewal, to replenish the MuSC pool (Okafor et al., 2023). An increase in autophagy flux has been observed during MuSC activation, both in isolated MuSCs and in muscles of LC3–GFP transgenic mice, and is further elevated during MuSC proliferation (Campanario et al., 2020; Tang and Rando, 2014). Given that autophagy provides cells with both nutrients and energy, its upregulation likely supports the energy demands of MuSC activation and proliferation. Indeed, activated and proliferating MuSCs purified from muscles exhibit higher mitochondrial activity and adenosine triphosphate (ATP) production than do quiescent MuSCs, suggesting that autophagy supplies the necessary cellular components for energy production (Rodgers et al., 2014). The inhibition of autophagy impedes MuSC activation following muscle damage. For example, ATG16L1 facilitates the elongation and closure of the autophagosomal membrane by promoting the lipidation of light chain 3 (LC3)/ATG8 (also known as MAP1LC3) (Mizushima et al., 2003). In *Atg16l1*-null mice, in which autophagy is present but reduced, a higher proportion of quiescent MuSCs (*Pax7^+^/Myod1^−^*) and a lower proportion of muscle precursors (*Pax7^+^/Myod1^+^*) or myoblasts (*Pax7^−^/Myod1^+^*) is observed compared to that in wild-type mice (Paolini et al., 2018). Similarly, autophagy inhibition in purified MuSCs from mouse muscles, induced by chemical inhibitors, such as 3-methyladenine (3-MA; Box 1) or chloroquine (Box 1), or by small interfering RNAs (siRNAs) that target *Atg5* and *Atg7*, significantly impairs MuSC activation, reducing ATP production and mitochondrial activity (Tang and Rando, 2014). These effects can be reversed by supplementing the culture media with exogenous sodium pyruvate (Tang and Rando, 2014). These findings suggest that increased autophagy meets the biological energy demands of MuSC activation and proliferation. However, recent studies indicate that hyperactive autophagy can also impair the proliferative capacity of MuSCs. The overactivation of autophagy has been observed in MuSCs differentiated from induced pluripotent stem cells (iPSCs) derived from patients with myotonic dystrophy type 1 (DM1) (Box 1), in which muscleblind-like 1 (*MBNL1*) expression is reduced (Song et al., 2020). MuSCs with elevated autophagy levels had proliferation defects, and the inhibition of autophagy partially restored MuSC proliferation (Song et al., 2020). Our research group's findings have also shown that the upregulation of autophagy impairs MuSC proliferation, underscoring the importance of fine-tuning autophagy in MuSCs (Goh et al., 2024).

### Differentiation and fusion

Autophagy has been shown to be essential for myogenic differentiation and fusion. During myoblast differentiation, autophagy is enhanced by increased levels of ATG7, Unc-51-like kinase 1 (ULK1), beclin 1 (BECN1) and LC3 (Box 2), and this enhancement persists throughout the differentiation process of C2C12 myoblasts *in vitro* (Baechler et al., 2019; Call et al., 2017; Fortini et al., 2016). An *in vivo* study using LC3–GFP mice has further confirmed that autophagy is continuously activated during MuSC activation and differentiation (Tang and Rando, 2014).

**Box 2. Autophagic machinery**

Under conditions of cellular stress, autophagy is initiated by the activation of the Unc-51-like kinase 1 (ULK1) complex, which consists of ULK1, FIP200, ATG13 and ATG101. This complex phosphorylates and recruits other proteins to the pre-autophagosomal structure to induce phagophore formation (Chen et al., 2008; Tang and Chen, 2011; Tang et al., 2023, 2011). Following the initiation of autophagy, the class III phosphatidylinositol 3-kinase complex 1 (PI3KC3-C1) generates phosphatidylinositol 3-phosphate (PtdIns3P), which recruits WD-repeat protein interacting with phosphoinositides (WIPIs) and the ATG8/LC3 conjugation machinery. WIPI2 interacts with ATG16L, facilitating the recruitment of the ATG12–ATG5–ATG16L complex, which promotes LC3 lipidation and accelerates autophagosome formation (Dooley et al., 2014). Autophagosomes engulf cellular components and transport them to lysosomes for degradation (Tang et al., 2018, 2021; Wang et al., 2021). The LC3 conjugation system plays a key role in this process, in which ATG7, an E1-like enzyme, transfers LC3-I to the E2-like enzyme ATG3, which interacts with ATG12 on the complex (Chen et al., 2012; Dooley et al., 2014). LC3-I is then conjugated with phosphatidylethanolamine (PE) to form LC3-II, a lipidated version crucial for autophagosome–lysosome fusion (Nguyen et al., 2016). The fusion of an autophagosome with a lysosome produces an autolysosome, in which cellular material is degraded and subsequently released into the cytoplasm for recycling or energy production.

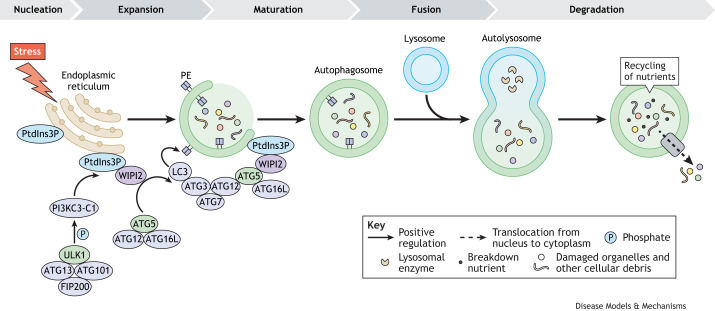

Whereas quiescent MuSCs primarily rely on glycolysis to meet their metabolic needs, myotubes are metabolically active cells that heavily depend on oxidative phosphorylation, which requires a substantial number of mitochondria (Pääsuke et al., 2016). Thus, the differentiation of MuSCs into mature myotubes necessitates mitochondrial renewal to support the increased energetic demands of skeletal muscle contractions. A previous study reported a significant upregulation of mitochondrial biogenesis at the onset of muscle regeneration in the tibialis anterior muscles of male Sprague-Dawley rats injected with bupivacaine, a myotoxic agent (Duguez et al., 2002). Additionally, mitochondria initially involved in glycolysis are recycled through mitophagy, a specific type of autophagy that selectively removes damaged or unwanted mitochondria (Sin et al., 2016). New mitochondria are then regenerated and produced (Sin et al., 2016). In differentiating MuSCs, in which the old mitochondrial network used for glycolysis is eliminated, autophagosomes and mitochondria are observed to be colocalized (Sin et al., 2016). As differentiation proceeds, autophagic flux decreases, and key factors associated with mitochondrial biogenesis, including PGC1α (also known as PPARGC1A) and OPA1, markedly increase, leading to enhanced mitochondrial content and to the formation of dense mitochondrial networks for oxidative phosphorylation (Baechler et al., 2019; Sin et al., 2016). The suppression of autophagosome formation, via the knockdown of *Atg5* in C2C12 myoblasts or 3-MA treatment, reduces the colocalization of mitochondria and autophagic puncta, impairs mitochondrial recycling, inhibits myoblast differentiation and causes myoblast apoptosis (Sin et al., 2016). Similar effects have been observed in C2C12 myoblasts treated with bafilomycin A1 (Baf-A1; Box 1), an inhibitor that blocks the fusion of autophagosomes with lysosomes (Sin et al., 2016). Consistently, in C2C12 myoblasts, the removal of BCL2 interacting protein 3 (BNIP3), a critical regulator of mitophagy, induces defects in myoblast differentiation and augments apoptosis (Baechler et al., 2019). These findings are further supported by parkin (PRKN)-mediated mitophagy (Esteca et al., 2020; Peker et al., 2018). PRKN is an E3 ubiquitin ligase that translocates to mitochondria upon loss of mitochondrial membrane potential to initiate mitophagy. siRNA-mediated knockdown of *Prkn* in C2C12 myoblasts results in the accumulation of dysfunctional mitochondria, leading to defects in muscle differentiation and fusion (Peker et al., 2018). Consistent with these *in vitro* results, muscles of the *Prkn* knockout mouse mutant showed muscle atrophy, including impaired mitochondrial function and smaller myofiber area (Esteca et al., 2020; Peker et al., 2018). Collectively, these studies indicate that the induction of autophagy during early MuSC differentiation is required for mitochondrial network reconstruction and myogenic differentiation.

By contrast, multiple studies have also shown that increased autophagy results in the suppression of muscle differentiation. The treatment of C2C12 myoblasts with Trillin, a small-molecule compound, has been shown to enhance autophagy levels and to reduce C2C12 differentiation (Dai et al., 2022). In mice, the intraperitoneal injection of spermidine enhanced autophagy in MuSCs, and spermidine-induced autophagy inhibited the myogenic differentiation of MuSCs, leading to muscle atrophy (Zhang et al., 2018). Consistent with these findings, our group has also shown that *Deaf1* knockdown induces autophagy in MuSCs, which inhibits muscle differentiation in both C2C12 myoblasts and purified mouse MuSCs (Goh et al., 2024). These studies indicate that autophagic activity needs to be strictly regulated during MuSC differentiation, because both the hyperactivation and impairment of autophagy lead to defects in muscle differentiation. Therefore, the precise regulation of autophagy during muscle regeneration is crucial for maintaining proper muscle function. Various transcription factors not only play key roles in regulating autophagy but also control MuSC renewal and differentiation. Understanding how these transcription factors influence autophagy will illuminate their roles in muscle regeneration and the development of muscular disorders.

## Transcription factor regulation of autophagy and muscular disorders

**Fig. 2.**
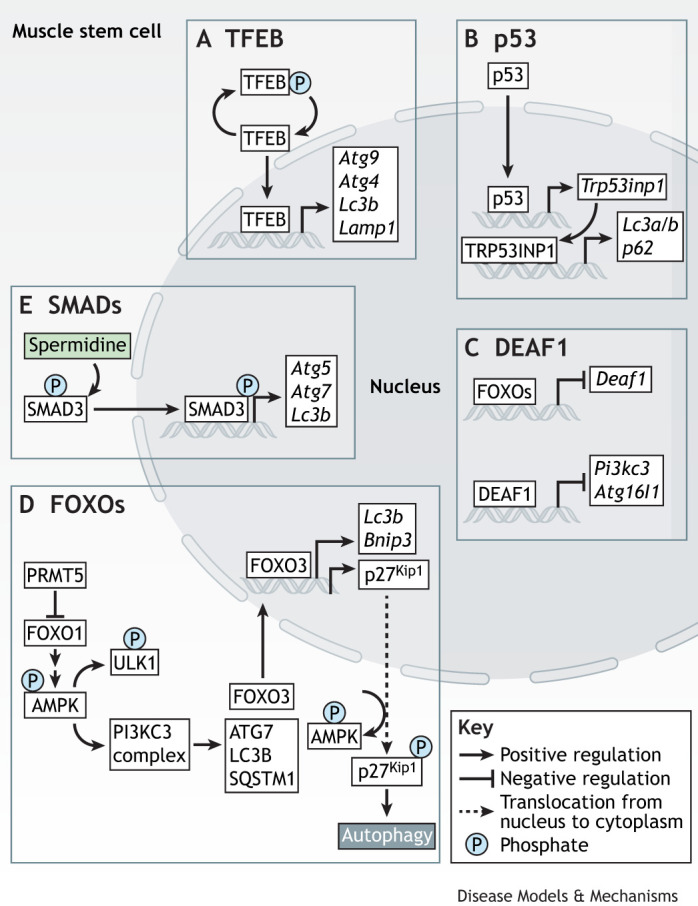
**Transcription factor regulation of autophagy in MuSCs.** (A-E) The transcription factors that regulate autophagy in MuSCs, including TFEB (A), p53 (B), DEAF1 (C), FOXOs (D) and SMADs (E). (A) Dephosphorylated TFEB translocates to the nucleus, activating autophagy-related genes including *Atg9*, *Atg4*, *Lc3b* and *Lamp1*. (B) p53 regulates autophagy genes *Lc3a/b* and *p62* (also known as *Sqstm1*) through the activation of TRP53INP1. (C) FOXO transcription factors regulate *Deaf1* expression. DEAF1, in turn, regulates the transcription of *Pi3kc3* and *Atg16l1*. (D) Inhibition of FOXO1 by PRMT5 initiates autophagy through the activation of autophagic machinery (Box 2), whereby phosphorylated AMPK leads to the phosphorylation of ULK1 and activates PI3KC3 complex, activating ATG7, LC3B, SQSTM1 and FOXO3. FOXO3 then translocates to the nucleus, resulting in increased autophagy through the activation of *Lc3b*, *Bnip3* and p27Kip1. (E) Spermidine activates SMAD3, which, upon phosphorylation, translocates to the nucleus and enhances the transcription of *Atg5*, *Atg7* and *Lc3b*. AMPK, AMP-activated protein kinase; ATG, autophagy related; *Bnip3*, BCL2 interacting protein 3; DEAF1, deformed epidermal autoregulatory factor 1; FOXO, Forkhead box class O; *Lamp1*, lysosomal-associated membrane protein 1; LC3, light chain 3; PI3KC3, class III phosphatidylinositol 3-kinase; PRMT5, protein arginine methyltransferase 5; SMAD, SMAD family member; SQSTM1, sequestosome 1; TFEB, transcriptional factor EB; TRP53INP1, tumor protein p53 inducible nuclear protein 1; ULK1, Unc-51-like kinase 1.

Transcription factors such as PAX3/PAX7 and myogenic regulatory factors play essential roles in regulating MuSC states and functions. However, the roles of other transcription factors in skeletal muscle homeostasis remain largely unexplored, with only a few having been studied. Autophagy, an essential mechanism for muscle regeneration, is regulated by multiple transcription factors during myogenesis (Fig. 2A-E). The mechanisms by which these transcription factors regulate autophagy during muscle regeneration and how their dysregulation leads to muscular disorders are discussed below.

### Forkhead box class O (FOXO)

The FOXO transcription factors, belonging to the Forkhead box protein family, are a highly conserved family of proteins, with orthologs characterized in worms, *Drosophila*, zebrafish, rodents and humans (Cheng, 2019). In mammals, there are four FOXO family members: FOXO1, FOXO3, FOXO4 and FOXO6. FOXO1, FOXO3 and FOXO4 are expressed ubiquitously, whereas FOXO6 is mainly expressed in the central nervous system (Sanchez et al., 2014). Post-translational modifications can modulate FOXO protein activity. For example, in the presence of insulin, the phosphatidylinositol 3-kinase (PI3K; also known as PIK3)–AKT/protein kinase B (PKB; also known as AKT1) pathway can be activated, leading to the phosphorylation of FOXOs, promoting their cytoplasmic retention, enhancing their degradation and inhibiting their functions as transcription factors (Sanchez et al., 2014). Interestingly, FOXO transcription factors also play critical roles in regulating autophagy, as they have been shown to bind to promoter regions of autophagy genes to modulate autophagy (Cheng, 2019; Goh et al., 2024). In addition to autophagy, FOXO factors regulate another essential clearance mechanism, the ubiquitin–proteasome system, which also plays a critical role in the removal of misfolded and damaged proteins (Sandri et al., 2004). Together, FOXO transcription factors play vital roles in cellular homeostasis in multiple tissues, including the liver, heart, neuronal cells and skeletal muscles (Cheng, 2019).

Given the roles of FOXOs in autophagy and skeletal muscle homeostasis, it is of interest to investigate the autophagy-regulating roles of FOXO transcription factors in the context of myogenesis. In a *Foxo1/3/4* muscle-specific knockout mouse model, the loss of these Foxo genes in the skeletal muscle of mice effectively protected their muscles from wasting under conditions of fasting or denervation, as autophagy was inhibited. This was evidenced by decreased LC3 lipidation, and by attenuated vesicle formation, in *Foxo1/3/4^−/−^* mice compared to that in controls (Milan et al., 2015). Significantly, another study has shown that FOXO3 regulates the expression of autophagy-related genes, such as *Map1lc3a* and *Bnip3*, in skeletal muscles to mediate muscle atrophy (Mammucari et al., 2007). In mouse models that mimic human muscle atrophy in the context of different diseases and conditions, such as denervation, cachexia (Box 1), starvation or sepsis, FOXO3 and FOXO4 upregulate macroautophagy and youth optimizer (MYTHO; also known as PHAF1), a newly identified regulator of muscle autophagy, to drive autophagic processes and promote muscle atrophy (Leduc-Gaudet et al., 2023). Mechanistically, FOXO3/4-mediated expression of MYTHO promotes autophagosome formation by binding to WIPI2 and BCAS3, facilitating the autophagic process (Leduc-Gaudet et al., 2023). In mice, intramuscular injections of adeno-associated virus (AAV) to knock down *Mytho* results in severe myopathic defects, attributed to impaired autophagy (Leduc-Gaudet et al., 2023). Collectively, these studies underscore the critical role of FOXO transcription factors in regulating autophagy and muscle atrophy, positioning them as key molecular targets for therapeutic intervention in muscle-wasting diseases.

Autophagy in MuSCs is also regulated by the FOXOs. The importance of FOXOs in MuSCs came to light when enriched transcription factor-binding motifs were compared between quiescent and primed MuSCs (Garcia-Prat et al., 2020). In this study, quiescent MuSCs were defined as being CD34^High^ and more stem-like. These cells showed enriched occupancy of the FOXO family DNA recognition motif in expressed genes compared to primed quiescent MuSCs, which were defined as being CD34^Low^ and as having fewer stemness properties (Garcia-Prat et al., 2020). Additionally, FOXO3 showed increased nuclear localization in genuine quiescent MuSCs compared to primed quiescent MuSCs. The depletion of FOXOs in the quiescent MuSCs also prompted their premature activation and reduced their regenerative capabilities, highlighting a key role for FOXOs in maintaining quiescent MuSCs (Garcia-Prat et al., 2020). The importance of FOXOs in mediating autophagy in MuSCs has also been highlighted in several studies. FOXO3 drives autophagy by binding to the promoters of autophagy-related genes (Bowman et al., 2014). In C2C12 mouse myoblasts, FOXO3 occupies the promoters of genes such as GABA type A receptor associated protein like 1 (*Gabarap1l*) , *Map1lc3b* and *Atg12* during starvation, enhancing autophagy. However, when nutrients are reintroduced, FOXO3 is displaced by FOXK1, a member of the Forkhead transcriptional family, which recruits the SIN3A–HDAC complex to repress gene expression. The study suggests that FOXK1 and FOXO3 compete for the same binding sites on these promoters, with overexpression of FOXK1 reducing FOXO3 binding and autophagy (Bowman et al., 2014). Importantly, the activities of FOXK1 and FOXO3 in regulating autophagy are modulated by nutrient availability and mammalian target of rapamycin complex 1 (mTORC1) signaling. In nutrient-rich conditions, mTORC1 activation promotes FOXK1 nuclear import, leading to autophagy gene repression. By contrast, starvation inhibits mTORC1, sequestering FOXK1 in the cytosol, while AKT inhibition promotes the nuclear localization of FOXO3, driving autophagy gene expression. Thus, mTORC1 integrates nutritional cues, and the competition between FOXK1 and FOXO3 ensures autophagic homeostasis in MuSCs (Bowman et al., 2014).

In another study on protein arginine methyltransferase 5 (PRMT5), an enzyme that catalyzes arginine methylation on target proteins and regulates MuSC proliferation and muscle regeneration, it was shown that PRMT5 modulates FOXO1 to mediate autophagy (Kim et al., 2023). Muscle-specific deletion of *Prmt5* in mice inhibited the proliferation of MuSCs and induced their differentiation. In the absence of PRMT5, FOXO1 levels were elevated, and it was discovered that PRMT5 regulates FOXO1 protein stability by methylating and destabilizing it, with this methylation occurring only in the nucleus of MuSCs. However, cytoplasmic accumulation of FOXO1 activated autophagy, depleted lipid droplets, and disrupted MuSC metabolism and homeostasis (Kim et al., 2023). The study further showed that FOXO1 interacts with ATG7 in the cytoplasm, but not in the nucleus, of C2C12 myoblasts. When FOXO1 was retained in the cytoplasm owing to a mutation in its DNA-binding domain, autophagy levels increased. Notably, pharmacological inhibition of autophagy rescued MuSC defects in the muscle-specific *Prmt5* knockout mice (Kim et al., 2023). These findings highlight the importance of tightly regulating FOXO levels and activity to maintain basal autophagy levels for MuSC homeostasis.

In alcohol consumption-induced muscle atrophy, ethanol has been shown to impair protein synthesis and to induce protein degradation in C2C12 mouse myoblasts via the FOXO1–AMPK–tuberin (TSC2)–mTORC1 pathway (Hong-Brown et al., 2017). Significantly, the addition of ethanol to C2C12 myoblasts activates AMPK and phosphorylates the S555 and S757 residues of ULK1, leading to increased ATG14 and PI3KC3, thus increasing autophagosome formation and autophagic flux, as evidenced by increased LC3 lipidation (Hong-Brown et al., 2017). The knockdown of AMPK or the inhibition of FOXO1 in these cells attenuated the phosphorylation of ULK1 at both S555 and S757, which decreased ATG7 and LC3-II levels and inhibited autophagy, rescuing the ethanol-induced C2C12 myoblast defects (Hong-Brown et al., 2017).

In the context of aging, one study has shown that, in aged MuSCs isolated from mice, AMPK and its downstream target p27^Kip1^, a cyclin-dependent kinase inhibitor, were hypophosphorylated, sequestering p27^Kip1^ in the nucleus. This was associated with increased autophagy and apoptosis in aged MuSCs, leading to their dysfunction (White et al., 2018). To ameliorate this effect, the authors treated the aged MuSCs with AICAR, an AMP analog, to activate AMPK. Activated AMPK phosphorylates p27^Kip1^, mediating its translocation from the nucleus to the cytoplasm and promoting autophagy, preventing senescence and apoptosis in the aged MuSCs, and thus improving MuSC function (White et al., 2018). Another study demonstrated that FOXO3 overexpression in MuSCs increased p27^Kip1^ expression (Rathbone et al., 2008). These findings highlight a potential epistatic mechanism in which FOXO3 mediates p27^Kip1^ expression, and the cytoplasmic localization of p27^Kip1^, which is dependent on AMPK-mediated phosphorylation, then promotes autophagy, maintaining the proliferative potential of aged MuSCs. Interestingly, the latter study showed that adenovirus-mediated overexpression of wild-type FOXO3 in MuSCs under normal conditions inhibited MuSC proliferation instead, increasing p27^Kip1^ expression (Rathbone et al., 2008). Thus, the regulatory balance between FOXO3, p27^Kip1^ and AMPK is crucial for MuSC homeostasis, particularly in aging, whereby disruption of these pathways could contribute to muscle stem cell dysfunction and impaired regeneration.

Taken together, these studies highlight the importance of regulating FOXO levels in MuSCs under different contexts. Under normal conditions, increased FOXO levels lead to increased autophagy, which in turn leads to MuSC homeostatic defects. However, under conditions in which autophagy is beneficial, such as in aging or alcohol-induced protein aggregation, increased FOXO levels drive increased autophagy, thus helping MuSCs to achieve an improved metabolic balance to maintain their proliferative and regenerative potential.

### SMAD family members (SMADs)

The SMADs are transcription factors in the transforming growth factor-β (TGF-β) signaling pathway, and transmit extracellular signals from TGF-β ligands to the nucleus. Ligands, including TGF-βs, activins and growth differentiation factors (GDFs), such as myostatin, bind to their respective receptors and initiate the receptor-mediated phosphorylation of SMAD2/3. The binding of bone morphogenic proteins (BMPs) or other GDFs results in the phosphorylation of SMAD1, SMAD5 and SMAD8. These phosphorylated SMADs then form complexes with SMAD4, translocating into the nucleus to modulate downstream gene expression (Goodman and Hornberger, 2014). Notably, the inhibition of GDF8 (also known as myostatin) leads to significant skeletal muscle hypertrophy in *Gdf8*-null mutant mice (McPherron et al., 1997). Elevated myostatin levels are often associated with increased SMAD3 phosphorylation (Langley et al., 2002) and are implicated in conditions such as cancer cachexia, in which muscle wasting occurs (Bajpai et al., 2020; Song et al., 2019; Zimmers et al., 2002). These findings underscore myostatin's role as a negative regulator of skeletal muscle mass, implicating SMAD signaling in this regulatory process and sparking interest in the roles of SMADs in muscle regulation.

*Smad3-*null mice exhibit decreased muscle mass and marked skeletal muscle atrophy, partially due to muscle regeneration defects (Ge et al., 2011). The MuSCs purified from the muscles of *Smad3*-null mice showed a reduced propensity for self-renewal, while *Smad3*-deficient myoblasts exhibited impaired differentiation and fusion, forming atrophied myotubes (Ge et al., 2011). These findings suggest that SMAD3 signaling is crucial for MuSC function and myogenic differentiation. Interestingly, when wild-type Wistar rats are intraperitoneally injected with SB431542, a TGF-β–SMAD pathway inhibitor, SMAD2/3 phosphorylation is reduced and the numbers of *Pax7^+^/Myod1^–^* and *Pax7^+^/Myod1^+^* cells are increased. This suggests that TGF-β–SMAD signaling inhibits MuSC activation and proliferation (Nikooie et al., 2020). Discrepancies between the results of Ge et al. (2011) and Nikooie et al. (2020) could stem from the distinct roles of SMADs and phosphorylated SMADs. Although the absence of SMAD3 impairs MuSC function and differentiation, inhibiting SMAD2/3 phosphorylation might stimulate MuSC activation. However, the findings from Nikooie et al. (2020) were not further validated using mouse genetic models, and the systemic effects of SB431542 injection on muscles or other tissues might have influenced the results.

Additionally, SMAD-mediated autophagy plays a critical role in muscle function. In *Drosophila*, Activin signaling activates the Smox transcription factor (ortholog of SMAD2/3 in humans) to directly target and inhibit the transcription of *Autophagy-specific gene 8a* (*Atg8a*), thus suppressing autophagy in *Drosophila* muscles (Bai et al., 2013). The accumulation of misfolded protein aggregates is a hallmark of aged muscles, while reduced Activin signaling enhances autophagy and improves protein homeostasis in aged flies (Bai et al., 2013). In mice, spermidine treatment increases the binding of SMAD3 to autophagy gene promoters and decreases its binding to myogenic genes, thus enhancing autophagy in MuSCs while blocking myogenic differentiation and causing muscle atrophy (Zhang et al., 2018). These findings suggest that autophagy acts downstream of SMAD signaling, influencing muscle protein homeostasis and differentiation. Overall, an improved understanding of the interplay between the SMAD–autophagy axis and SMAD-mediated myogenic gene transcription will be crucial for elucidating the role of SMADs in MuSC regulation. Further research is therefore needed to fully comprehend how these mechanisms contribute to muscle function and dysfunction, particularly in pathological conditions, such as muscle wasting, and in aging.

### Kruppel-like factors (KLFs)

The KLFs constitute a family of 18 zinc finger transcription factors that regulate diverse biological processes, including proliferation, differentiation and tissue development (Oishi and Manabe, 2018). Several KLFs have key roles in muscle biology, influencing MuSC functions, and responses to atrophy and regeneration (Oishi and Manabe, 2018). KLF15 is a key regulator of lipid homeostasis in skeletal muscles, maintaining metabolic homeostasis under both basal conditions and in response to nutrient overload (Fan et al., 2021). In mice with muscle-specific deletion of *Klf15*, the expression of genes involved in lipid uptake, mitochondrial transport and utilization, is significantly reduced. This leads to elevated circulating lipid levels, underscoring its importance in regulating lipid metabolism (Fan et al., 2021). The upregulation of KLF15 in hyperglycemia (Box 1) leads to the activation of genes involved in protein catabolism and muscle atrophy, highlighting KLF15 as a potential therapeutic target for managing skeletal muscle decline in diabetes mellitus (Hirata et al., 2019). KLF6 expression in C2C12 myoblasts is induced by TGF-β–SMAD3 signaling, promoting myoblast proliferation; its depletion enhances myogenic differentiation (Dionyssiou et al., 2013). Similarly, KLF7 expression in purified MuSCs and in C2C12 myoblasts is positively regulated by TGF-β and Notch pathways, and is crucial for maintaining MuSC quiescence through p21 (also known as CDKN1A) activation (Wang et al., 2016). KLF2 and KLF4 are upregulated during C2C12 myoblast differentiation, in which they play essential roles in muscle cell fusion (Sunadome et al., 2011). KLF4 specifically promotes myoblast fusion by activating myomixer (Box 1), a muscle-specific micropeptide that regulates the fusion of myoblasts during myogenesis (Bi et al., 2017), and regulates proliferation through P57 (also known as CDKN1C)-mediated cell cycle arrest (Cai et al., 2023). KLF5 is elevated during myoblast differentiation and muscle regeneration, both in isolated MuSCs cultured in differentiation medium and in mice following cardiotoxin injection. KLF5 binds to *MyoD* and Mef2 genes to control MuSC differentiation. *Klf5* knockout in C2C12 cells or MuSC-specific *Klf5* knockout in mice impairs muscle regeneration and myotube formation (Hayashi et al., 2016). Together, these studies demonstrate the critical roles of KLFs in muscle regeneration and atrophy.

Emerging evidence suggests that KLFs also regulate autophagy. Chromatin immunoprecipitation (ChIP) assays performed on chromatin extracted from murine smooth muscle cells reveal that KLF2 and KLF4 bind to multiple autophagy-related genes and activate their expression in smooth muscle cells (Salmon et al., 2019). Although KLFs have been implicated in autophagy regulation across various cell types and species (Laha et al., 2019; Mei et al., 2022; Salmon et al., 2019), their specific role in regulating autophagy in MuSCs remains unexplored. Future studies are therefore needed to elucidate how KLFs influence autophagy dynamics during muscle regeneration. Understanding these mechanisms could unveil new therapeutic avenues for managing muscle disorders in which autophagy dysregulation plays a key role.

### Transcriptional factor EB (TFEB)

TFEB, a member of the microphthalmia family (Cui et al., 2023), is a basic helix-loop-helix leucine zipper (bHLH-Zip) transcription factor that is primarily localized in the cytoplasm under normal conditions. In response to a variety of stimuli, including starvation and lysosomal stress, TFEB undergoes dephosphorylation and translocates into the nucleus, where it activates target gene transcription (Gebrie, 2023). TFEB exerts its regulatory role by binding to the coordinated lysosomal expression and regulation (CLEAR) motif in lysosomal gene promoters (Wang et al., 2023b) and to the Ephrussi boxes (E-boxes) in autophagy gene promoters (Franco-Juarez et al., 2022), thereby governing lysosomal biogenesis and autophagy processes. Its pivotal functions in these pathways establish TFEB as a central regulator in autophagy–lysosomal mechanisms.

In skeletal muscle and MuSCs, TFEB plays a critical role in maintaining cellular health by regulating autophagy and lysosomal biogenesis. As a master regulator of these processes, TFEB controls the expression of genes that promote lysosomal function and autophagic flux, both of which are essential for clearing damaged organelles and proteins. This regulatory capacity has made TFEB a key therapeutic target for several myopathic conditions, such as Pompe disease (Box 1), which is characterized by glycogen accumulation due to acid α-glucosidase deficiency, and Duchenne muscular dystrophy (DMD; Box 1), in which muscle atrophy results from autophagy dysregulation. In mouse models of Pompe disease, TFEB overexpression improved muscle pathology and function by enhancing autophagic flux and reducing glycogen buildup. Similarly, TFEB gene transfer improved skeletal muscle pathology in iPSCs derived from late-onset Pompe disease, which were induced to differentiate into skeletal muscle cells (Sato et al., 2016; Spampanato et al., 2013). In a mouse model of DMD, reduced TFEB localization in muscle nuclei correlated with the decreased expression of autophagy-related genes (Nakashima et al., 2024). Additionally, in MuSC-specific androgen receptor and estrogen receptor 2 double knockout mice, muscle regeneration was attenuated, and significant increases in DNA damage and senescent markers were observed, in MuSCs (Kim et al., 2021). Such increased DNA damage and senescence in MuSCs were attributed to decreased autophagic clearance due to reduced TFEB expression in MuSCs. This study further demonstrated that transduction of TFEB into MuSCs significantly increased muscle mass in old mice, highlighting the role of TFEB in promoting autophagosome clearance, which is crucial for preventing senescence and supporting muscle regeneration (Kim et al., 2021).

In another study, knockdown of palmitoyl protein thioesterase (*Ppt1*), a critical molecule in the mTORC1–TFEB signaling pathway (Box 1), inhibited muscle differentiation in C2C12 myoblasts (Yun et al., 2020). The inhibition of PPT1 in this model prevented mTORC1 activation, activating TFEB and causing an accumulation of autophagic flux, impairing muscle fiber formation (Yun et al., 2020). Interestingly, one study found that the activation of TFEB in mice by rapamycin treatment was unable to correct autophagy inhibition or increase lysosome number to rescue muscle disease caused by deficient inositol polyphosphate 5-phosphatase (INPP5K), a membrane-bound phosphoinositide that is vital for several stages of autophagy (McGrath et al., 2021). In this study, the loss of INPP5K in muscle inhibited autophagy and depleted lysosomes, and myoblasts deficient in INPP5K had enlarged autolysosomes, defective lysosome recycling and impaired autophagic lysosome reformation, a process that was not rescued by TFEB activation (McGrath et al., 2021). Instead, INPP5K was found to regulate autophagic lysosome reformation, an autophagy-dependent process that repopulates lysosomes and that causes muscle disease when it is dysregulated (McGrath et al., 2021). This is surprising given the vital role of TFEB in lysosome biogenesis, which was unable to compensate for lysosome homeostasis in the presence of defective autophagic lysosome reformation. This finding suggests that the context in which TFEB-mediated autophagy is targeted in MuSCs to treat muscle disease should be carefully considered, and should involve a thorough understanding of the mechanisms that are causing the muscle-wasting phenotype.

### Nuclear factor-kappa B (NF-κB)

NF-κB transcription factors have central roles in regulating MuSCs and have been linked to muscle wasting induced by cancer cachexia and sarcopenia (Box 1) (Bar-Shai et al., 2005; He et al., 2013). The mammalian NF-κB transcription factor family consists of five proteins [RELA, RELB, c-REL (also known as REL), p105/p50 (NF-κB1) and p100/52 (NF-κB2)], which can form homodimers and heterodimers, and which bind to promoter and enhancer regions to modulate gene expression (Yu et al., 2020). In the classical signaling pathway, IκB (also known as NFKBI) proteins bind to and suppress NF-κB proteins. Growth factors or cytokines can alleviate this repression by activating an IKK complex, which phosphorylates IκB, leading to its ubiquitination and degradation, and to the activation of NF-κB. Activated NF-κB translocates to the nucleus and induces target gene expression. In the non-canonical NF-κB pathway, ligands, such as tumor necrosis factor (TNF), super family members B-cell activating factor (BAFF; also known as TNFSF13B) (Claudio et al., 2002), CD40 ligand (CD40L; also known as CD40LG) (Coope et al., 2002), granulocyte-macrophage colony stimulating factor (GM-CSF; also known as CSF2) and M-CSF (also known as CSF1) (Jin et al., 2014), bind to and activate their receptors, triggering NF-κB inducing kinase (NIK; also known as MAP3K14) to phosphorylate and activate the IKKα complex, which then phosphorylates NF-κB p100. This leads to the processing and liberation of the NF-κB p52/RelB active heterodimer, which translocates to the nucleus and induces target gene expression (Liu et al., 2017).

NF-κB promotes myoblast proliferation and inhibits myogenic differentiation in the C2C12 myoblast cell line by upregulating cyclin D1 expression and hyperphosphorylation of retinoblastoma protein (pRb; also known as RB1) (Guttridge et al., 1999). As a tumor suppressor, pRb normally inhibits the G1–S transition to block cell cycle progression. However, NF-κB-driven hyperphosphorylation inactivates pRb, releasing its inhibitory control and allowing the cell cycle to proceed, thereby promoting proliferation. This pathway simultaneously suppresses myogenic differentiation, as cell cycle exit is essential for myoblasts to differentiate into mature muscle cells (Guttridge et al., 1999). Consistently, another study found that NF-κB binds to the promoter of the transcription factor YinYang1 (YY1) and stimulates its expression (Wang et al., 2007). NF-κB-induced YY1 then suppresses the expression of multiple myofibrillar genes (Wang et al., 2007), indicating that NF-κB inhibits MuSC differentiation. Interestingly, in a mouse cancer cachexia model bearing Colon-26 (C-26) tumors, the activation of NF-κB increases PAX7 expression and impairs MuSC differentiation (He et al., 2013). Satellite cell-specific increases in NF-κB activity have also been shown to lead to skeletal muscle regenerative defects in mice (Oh et al., 2016), and the inhibition of NF-κB has been shown to enhance myogenic potential, resulting in improved muscle regeneration in aged mice (Lu et al., 2012; Oh et al., 2016). These studies together suggest that NF-κB functions as a negative regulator of muscle regeneration, and that NF-κB overactivation contributes to muscle wasting during cancer cachexia or aging, highlighting NF-κB as a potentially druggable target to ameliorate muscle wasting in these two conditions. However, reported findings have also shown that NF-κB promotes myogenesis. Insulin growth factor-II (IGF-II)-activated NF-κB has been shown to stimulate the myogenic signaling pathway and to induce myogenesis in rat L6E9 myoblast cells (Kaliman et al., 1999). The discrepancy in these findings could arise from a switch between the classical and non-canonical NF-κB signaling pathway, with each playing distinct roles in regulating myogenesis, as demonstrated in cultured mouse myoblasts (Bakkar et al., 2008). The classical NF-κB pathway is activated exclusively in proliferating myoblasts and is not involved in their differentiation into multinucleated myotubes. By contrast, during myogenic differentiation, the non-canonical NF-κB pathway becomes active and enhances mitochondrial biogenesis in mice (Bakkar et al., 2012). Furthermore, another study in a mouse model has shown that activation of the non-canonical NF-κB signaling pathway promotes myoblast fusion during myogenesis (Straughn et al., 2019). This evidence suggests that the balance between these two pathways is crucial for understanding muscle regeneration and differentiation.

NF-κB has also been shown to activate autophagy in skeletal muscles. NF-κB activation induced by tumor necrosis factor-like weak inducer of apoptosis (TWEAK; also known as TNFSF12), a well-known NF-κB-dependent inducer of atrophy in skeletal muscles, has been shown to stimulate the transcription of autophagy-related genes, such as *Atg5*, *Becn1* and *Lc3b*, in C2C12-differentiated myotubes, consequently inducing skeletal muscle atrophy (Dogra et al., 2007). In C2C12 myotubes, TNFα-induced *Bnip3*, *Lc3a* and *Lc3b* mRNA expression and autophagy activation can be consistently reversed by suppressing NF-κB activity, indicating that NF-κB is required for autophagy during TNFα-induced muscle wasting (Yi et al., 2022). These studies highlight that the induction of NF-κB signaling could activate autophagy in muscle. Although NF-κB has been shown to induce autophagy in muscles, the roles of NF-κB in regulating autophagic flux in MuSCs remain unclear. Given that NF-κB can either positively or negatively influence MuSC regeneration, and considering that autophagy plays a crucial role in this regeneration process, the autophagy activity regulated by NF-κB could be vital for myogenesis. Understanding how NF-κB impacts autophagy in MuSCs could provide valuable insights into the mechanisms underlying muscle development and repair.

### p53

The well-characterized tumor suppressor p53 (also known as TRP53), often lauded as the ‘guardian of the cellular genome’, is a critical player in the cellular response to stress adaptation. In normal physiological conditions, p53 levels are tightly regulated by MDM2, an E3 ubiquitin ligase that can promote the proteasomal degradation of p53 (Chibaya et al., 2021). However, when cells are exposed to stress, p53 is post-translationally modified and stabilized, then translocates to the nucleus and promotes gene transcription. p53 can also modulate cellular responses to stress in a transcription-independent manner (Ho et al., 2019), and mediates various downstream pathways that regulate cell cycle arrest, apoptosis, senescence and autophagy (Wang et al., 2023a; White, 2016).

The roles of p53 in autophagy have been extensively studied across multiple cell and tissue types. For example, it has known roles in mitophagy; nuclear-localized p53 has been shown to downregulate PINK1, a key protein in mitophagy regulation, in human neuroblastoma cells (Goiran et al., 2018). In other studies, mutant p53 has been shown to promote mitophagy, promoting survival in non-small cell lung cancer (Wang et al., 2022) and in mouse embryonic fibroblasts (Gao et al., 2022). In skeletal muscles, the loss of p53 has minimal impact under normal conditions but becomes significant during muscle disuse. In a mouse model of muscle disuse, p53 was identified as being a crucial regulator of immobilization-induced muscle atrophy, with no observable effects in mice that maintained regular mobility (Fox et al., 2014). The ablation of *Trp53* worsened muscle decline in a chronic skeletal muscle disuse model due to impaired mitophagy, which disrupted mitochondrial quality control and reduced mitochondrial function (Memme et al., 2022). Interestingly, in a separate study, breast cancer tumors were shown to secrete extracellular vesicles (Box 1) containing miR-122-5p, which targets and reduces p53 levels in myocytes, ultimately leading to muscle atrophy via mitochondrial dysfunction (Ruan et al., 2023). These findings highlight the critical role of p53 in maintaining muscle health during conditions of stress, such as disuse or cancer, through its regulation of mitochondrial function. Disruption of p53, whether through genetic deletion or tumor-secreted factors, compromises mitochondrial quality control and contributes to muscle degeneration. Therefore, p53 represents a potential therapeutic target for preventing muscle atrophy in conditions involving prolonged disuse or cancer-related cachexia.

In the context of MuSCs, MuSCs without p53 have attenuated levels of basal autophagy, inhibiting their terminal differentiation into mature myocytes, as shown in MuSCs purified from mouse muscles (Fortini et al., 2016). Interestingly, mitochondrial biogenesis is also impaired upon loss of p53 in these myoblasts (Fortini et al., 2016). Another study found that autophagy dysregulation in the muscle precursor cells of patients with type 2 diabetes (T2DM) leads to impaired myogenesis (Henriksen et al., 2019). In this study, the authors isolated muscle precursor cells from individuals with and without T2DM. Upon inducing differentiation, muscle precursors from individuals with T2DM showed lower protein levels of ATG7 and downregulated levels of tumor protein p53 inducible nuclear protein 1 (*TP53INP1*) mRNA compared to those in precursors from unaffected individuals. This suggests that the autophagy response during myogenesis is impaired in individuals with T2DM, potentially contributing to their compromised regenerative capacity. *TP53INP1* is a p53 target gene involved in the regulation of cell death and cell cycle arrest (Seillier et al., 2012), and when it was knocked down in healthy human muscle precursor cells, apoptotic markers increased. These findings indicate that p53 regulates apoptosis in an autophagy-dependent manner through TP53INP1 during myogenic differentiation (Henriksen et al., 2019). Taken together, these studies suggest that p53 has an important role in the regulation of autophagy, especially in MuSCs, as the disruption of p53 in basal conditions inhibited MuSC differentiation, while its ablation in muscles under basal conditions produced no significant muscle atrophy phenotypes compared to controls (Ebert et al., 2019; Memme et al., 2022; Stocks et al., 2017).

### Deformed epidermal autoregulatory factor 1 (DEAF1)

DEAF1 is an evolutionarily conserved transcription factor that binds to TTCG motifs in DNA (Huggenvik et al., 1998). Initially isolated in *Drosophila*, homologs of Deaf1 were subsequently discovered in humans, rats and monkeys (Jensik et al., 2004; LeBoeuf et al., 1998). Studies have shown that loss of DEAF1 function leads to neural tube defects in mice and to developmental arrest in *Drosophila* (Hahm et al., 2004; Veraksa et al., 2002). In humans, DEAF1 has been implicated in various neurodevelopmental disorders, autoimmune diseases (McGee et al., 2023; Michelson et al., 1999) and cancers (Manne et al., 2001).

Recently, a comprehensive RNA interference screening in *Drosophila* larval muscles identified Deaf1 as a novel regulator of muscle physiology (Graca et al., 2021). *Deaf1* depletion in *Drosophila* was found to increase myofiber size, whereas its overexpression induced muscle atrophy. These effects were attributed to the Deaf1-mediated regulation of glycolysis, although its direct targets in this context have yet to be identified (Graca et al., 2021).

In a recent study by our group, we explored the role of DEAF1 in regulating MuSC behavior in *Drosophila* and mice (Goh et al., 2024). Surprisingly, we found that manipulating Deaf1 expression in adult *Drosophila* MuSCs significantly affected MuSC numbers and impaired their myogenic differentiation (Goh et al., 2024). ChIP sequencing (ChIP-seq) analysis showed that DEAF1 binds to the promoter regions of PI3KC3 and ATG16L1, suppressing their expression. Consequently, inhibiting DEAF1-activated autophagy in MuSCs leads to cell death, whereas DEAF1 overexpression inhibits autophagy and induces protein aggregate formation, impairing muscle regeneration. Our research has also shown that FOXOs negatively regulate DEAF1, highlighting a conserved FOXOs–DEAF1–autophagy pathway from *Drosophila* to mammals (Goh et al., 2024). Interestingly, DEAF1 expression is elevated in MuSCs from aged mice and in muscles of patients with sarcopenia (Goh et al., 2024; Migliavacca et al., 2019). Reducing DEAF1 expression in these contexts reversed age-related muscle weakness associated with sarcopenia (Goh et al., 2024). Conversely, DEAF1 expression was reduced in MuSCs from cachectic mice and in the muscles of patients with cachexia (Goh et al., 2024; Narasimhan et al., 2021). Elevating DEAF1 levels in muscles rescued cancer-induced defects in muscle regeneration, underscoring the critical role of the FOXOs–DEAF1–autophagy pathway in muscle health and its implications in sarcopenia and cancer cachexia (Goh et al., 2024). These findings thus show that DEAF1 is a pivotal regulator in muscle biology, influencing MuSC behavior and muscle regeneration through modulation of autophagy. The intricate interplay between DEAF1, FOXOs and autophagy pathways underscores the potential of DEAF1 as a therapeutic target for conditions affecting muscle integrity and function.

## Conclusions and future perspectives

The intricate dynamics of MuSC behaviors reveal a complex interplay of signaling pathways that are crucial for promoting muscle repair and for maintaining MuSCs. Among these pathways, autophagy stands out as a key player in facilitating muscle regeneration. Restoring a balance in autophagy could provide a therapeutic means by which to enhance MuSC regeneration in aging or in muscular atrophic conditions. However, current autophagy modulators, such as rapamycin, an mTORC1 inhibitor, not only activate autophagy but also impact cellular growth, metabolism and survival. In fact, rapamycin has been shown to be toxic for muscles (Pallafacchina et al., 2002). To overcome this challenge, alternative approaches to modulate autophagy, such as dietary regulation, are being explored (Fiacco et al., 2016; Grumati et al., 2010). Strategies such as low-protein diets or periodic fasting have demonstrated the ability to reactivate autophagy, leading to improved myofiber regeneration and better outcomes for muscular dystrophy, albeit with reduced specificity (Fiacco et al., 2016; Grumati et al., 2010). Another potential option is the use of AAV vectors, such as AAV2 and AAV9, which specifically target skeletal muscle to deliver genes that regulate autophagy (Goh et al., 2024; Pankajakshan et al., 2012). Ongoing studies are delving into the transcriptional regulation of autophagy and have shown success in modulating autophagy levels in MuSCs and in muscles in mice using AAV to deliver transcription factors (Goh et al., 2024). Achieving a better understanding of the transcriptional cascade that governs the regulation of autophagy is crucial, as this cascade might influence biological processes independently of cellular nutrient status. Furthermore, identifying novel transcriptional modulators of autophagy, using computational strategies integrated with omics analysis data, could reveal new therapeutic targets. Manipulating these targets through AAV9 to enable selective modulation of autophagy types and levels in MuSCs could also provide exciting opportunities for understanding and treating muscle disorders, as well as for exploring innovative therapeutic outcomes.
